# Iduronic Acid in Chondroitin/Dermatan Sulfate Affects Directional Migration of Aortic Smooth Muscle Cells

**DOI:** 10.1371/journal.pone.0066704

**Published:** 2013-07-02

**Authors:** Barbara Bartolini, Martin A. Thelin, Lena Svensson, Giancarlo Ghiselli, Toin H. van Kuppevelt, Anders Malmström, Marco Maccarana

**Affiliations:** 1 Department of Experimental Medical Science, Lund University, Lund, Sweden; 2 Department of Experimental Medical Science, Immunology Section, Lund University, Lund, Sweden; 3 Glyconova Srl, Torino, Italy; 4 Department of Biochemistry, Nijmegen Center for Molecular Life Sciences, Radboud University Nijmegen Medical Center, Nijmegen, The Netherlands; University of Patras, Greece

## Abstract

Aortic smooth muscle cells produce chondroitin/dermatan sulfate (CS/DS) proteoglycans that regulate extracellular matrix organization and cell behavior in normal and pathological conditions. A unique feature of CS/DS proteoglycans is the presence of iduronic acid (IdoA), catalyzed by two DS epimerases. Functional ablation of DS-epi1, the main epimerase in these cells, resulted in a major reduction of IdoA both on cell surface and in secreted CS/DS proteoglycans. Downregulation of IdoA led to delayed ability to re-populate wounded areas due to loss of directional persistence of migration. DS-epi1−/− aortic smooth muscle cells, however, had not lost the general property of migration showing even increased speed of movement compared to wild type cells. Where the cell membrane adheres to the substratum, stress fibers were denser whereas focal adhesion sites were fewer. Total cellular expression of focal adhesion kinase (FAK) and phospho-FAK (pFAK) was decreased in mutant cells compared to control cells. As many pathological conditions are dependent on migration, modulation of IdoA content may point to therapeutic strategies for diseases such as cancer and atherosclerosis.

## Introduction

Proteoglycans (PGs) consist of glycosaminoglycan (GAG) chains attached to core proteins, and PGs can be found either in the extracellular space or bound to the cell membrane. Cell membrane-bound PGs may act as co-receptors and regulate biological processes such as proliferation, adhesion and migration, and these effects are mostly due to the PGs’ ability to interact and modulate the activity of growth factors, cytokines [1], [2] and integrins [3].

Two major types of GAGs, chondroitin/dermatan sulfate (CS/DS) and heparan sulfate (HS), are characteristic components of PGs. CS/DS chains are polymers consisting of repeated units of glucuronic acid (GlcA) or its epimer iduronic acid (IdoA) and *N-*acetyl-galactosamine (GalNAc). Conversion of GlcA to IdoA is catalyzed by dermatan sulfate epimerase 1 (DS-epi1) and 2 (DS-epi2) [4], [5]. IdoA residues confer flexibility to the polysaccharide [6], and their presence within the chain dictates the name dermatan sulfate. The epimerization of GlcA is variable along the chain and results in different domains, *i.e.* IdoA clusters, referred as IdoA blocks, and alternating IdoA/GlcA structures [5]. The action of DS-epi1 and 2 together with *O*-sulfotransferases [7], adding sulfate groups in 2-*O* position of IdoA/GlcA and 4-*O* and/or 6-*O* of GalNAc, produce a set of different structures that confer further complexity to the CS/DS chains. The IdoA regions are mainly 4-*O-*sulfated due to the strong stimulatory effect of dermatan sulfate 4-*O*-sulfotransferase 1 on IdoA formation [8].

CS/DS chains characterize PGs such as versican, aggrecan, decorin and biglycan, which are mainly secreted in the extracellular matrix (ECM) where they contribute to the structural organization of the tissue. Additionally, CS/DS chains can be present on the cell surface PGs, such as CS/DS/HS-PGs syndecan-1, -3 and -4 [2], [9] betaglycan [10], the part-time PG CD44 [11], NG2 [12] and integrin α_5_β_1_ [13]. Previous studies support the role of the CS/DS-HS-PG syndecans in signaling, adhesion, invasion and migration [2]. Specific CS/DS patterns induce different biological effects [14]. For example, recent studies showed that IdoA-containing structures are important for neurite outgrowth [15], [16], as well as for migration and proliferation of keratinocytes *in vitro* [17]. We have shown that cell surface IdoA-rich DS binds to hepatocyte growth factor, resulting in activation of the c-MET signaling pathway [1].

Here, we isolated aortic smooth muscle cells (AoSMCs) from wild-type (WT) and DS-epi1−/− mice previously generated [18] as an *in vitro* model system to further elucidate if IdoA in DS has a functional role in cellular processes such as migration, adhesion and cytoskeletal organization. We found that functional ablation of DS-epi1 in AoSMCs leads to decreased directional migration and reduced focal adhesion sites.

## Materials and Methods

### Materials

Collagenase type II was from Gibco. F12 medium and New-born Calf Serum (NBCS) were from Invitrogen. Superdex Peptide 10/300 GL, Superose 6 10/30, PD-10 columns, and ECL Plus reagent were from GE Healthcare. DE52 anion-exchange resin was from Whatman. ^35^SO_4_ (1,500 Ci/mmol) was from Perkin-Elmer. Chondroitinases ABC, B, AC-I, AC-II and heparitinase were from Seigakaku. Anti-HS mouse monoclonal antibodies 10E4, HepSS-1 and NAH46 from Seikagaku were purchased through AMSBIO UK. The anti-HS monoclonal antibody JM403 was a kind gift from Johan van der Vlag, Department of Nephrology Radboud University Nijmegen Medical Centre, The Netherlands. Anti-DS single chain variable fragment GD3A12, which recognizes IdoA and sulfated IdoA in DS, was produced and characterized as described in [19]. Rabbit (V4888) anti-tag VSV and the goat anti-mouse FITC-conjugated antibody were from Sigma. Anti-vinculin was from Sigma (cat. No. V4139), anti-FAK was from Sigma (cat. No. F2918; used for WB experiments) and from BD Transduction Laboratories (cat. No. 610088; used for TIRF images), anti-pY397 FAK was BD Transduction Laboratories (cat. No. 611806).

### Isolation of Aortic Smooth Muscle Cells (AoSMCs) and Establishment of Primary Culture

AoSMCs were prepared from 3 WT and 3 DS-epi1−/− mice (mixed C57BL6/Sv129 genetic background) of 5 weeks of age. Aortas were dissected and cleaned from adventitia. The remaining tissue was cut and digested with collagenase II 1 mg/mL for 3 hours at 37°C. Harvested cells were pooled for each genotype and seeded in T25 flasks with F12 medium supplemented with 10% NBCS, penicillin (100 µg/mL), and streptomycin (100 µg/mL) and grown for 3 days before changing the medium. At confluence, cells were split (passage 1). The genotype of isolated cells was confirmed again after 3 passages in culture by PCR with primers and conditions previously described [18].

The use of animals for research complied with national guidelines, and specific permit (M164-10) was issued by the Malmö-Lund animal care ethical committee. Mice were sacrificed by CO_2_ inhalation.

### Immunoblot Analysis

Cells were lysed and western blot anti-DS-epi1 was performed according to [18]. For SDS-PAGE analysis of labeled PGs, 20,000 dpm were loaded on a 4–12% gradient gel, transferred on a PVDF membrane and exposed to autoradiography film for 24 hours. Immunoblotting of FAK and pFAK were performed as previously described [1].

### Epimerase Assay

Epimerase activity was measured as previously described [5].

### Isolation of Metabolically Labeled GAG Chains from Medium

Cells were grown in flasks with F12 medium supplemented with 10% NBCS, and newly synthesized PGs were labeled by changing medium to sulfate-deprived MEM, 10% FBS, 1% glutamine, 1% PEST, and 100 µCi/mL of ^35^SO_4_. Labeled PGs and GAGs were subsequently isolated from medium and β-eliminated. HS was degraded, and purified CS/DS was analyzed according to Pacheco et al. [5].

### Flow Cytometry Analysis

Staining of IdoA-containing epitope on the cell surface was performed by flow cytometry as previously described [1]. Briefly, cells were incubated sequentially with phage display antibody against DS (GD3A12; 1∶80), rabbit anti-VSV tag (1∶400) and donkey anti-rabbit IgG 488 (1∶200; Jackson). A FACS-Calibur instrument integrated with Cell-Quest software (BD Biosciences) and Flowjo were used for analysis.

Analysis of HS cell surface structures was performed on cells harvested by detachment with DPBS without Ca/Mg, 0.2% Na_2_EDTA, pH 7.6, washed by centrifugation in DPBS, 1% BSA, 0.1% NaN_3_, pH 7.6 and resuspended in 250 µL of the same buffer (buffer A). Twenty-five µL of the cell suspension were incubated with 10 µL (20 ng) of the indicated mAb for 1 hr at 4°C. Ten µL of anti-mouse IgM fluorescein-conjugated antibody (50 ng) were then added and the samples incubated for an additional hour at 4°C. Finally the cells were recovered by centrifugation, washed twice and resuspended in buffer A and the associated fluorescence read on a GUAVA EasyCyte instrument. The generated FCS2 files were analyzed using the WinMDI 2.9 software.

### Immunocytochemistry

Immunostaining of cell surface IdoA was performed using anti-DS antibody (GD3A12) as previously described [1]. F-actin staining was performed using 488-phalloidin (A12379; Life Technologies) according to the manufacturer. Cells were analyzed using a Zeiss LSM 710 and Zeiss LSM 510 confocal scanning microscopes equipped with a 20× and 63× objectives.

### Cell Morphology Evaluation

WT and DS-epi1−/− AoSMCs were seeded in 6-well plates at a density of 4000 cells/cm^2^ and left at 37°C for 24 h. Cells were then rinsed with PBS, fixed and stained with crystal violet as reported previously [20]. Five different fields for each genotype were captured and analyzed for total area by ImageJ software.

### FAK qRT-PCR

Total RNA was extracted from WT and DS-epi1−/− AoSMCs with GeneJET RNA Purification kit (Fermentas K0731, Sweden) and the quality assessed on agarose gel. An amount of 100 ng was retrotranscribed by Maxima First Strand cDNA Synthesis Kit for RT-PCR (Fermentas K1641, Sweden) and qRT-PCR was performed using Maxima SYBR Green/ROX qPCR Master Mix (2×) (Thermo Scientific K0221), using final 300 nM of the following primers: mouse FAK forward 5′-TGTGATCGGTCGAATTGAAA-3′ and reverse 5′-TCCATCCTCATCCGTTCTTC-3′; mouse GADPH forward 5′-ACTCCACTCACGGCAAATTC-3′ and reverse 5′-TCTCCATGGTGGTGAAGACA-3′; cDNA was amplified in Stratagene M×3005P (Agilent Technologies), starting with an initial 10-min heating at 95°C, followed by 40 cycles at 95°C for 15 s, 55°C for 30 s and 72°C for 30 s. The data were analyzed with SDS 2.1 software (Applied Biosystems). The calculated threshold cycle (*C*_T_) values were normalized to the *C*_T_ value for mouse GADPH. Satisfactory efficiency of the reaction was assessed by dilutions of the cDNA.

### Wound Scratch Assay

AoSMCs were seeded in 6-well plates at a density of 8000 cells/cm^2^ and grown in F12 medium supplemented with 10% NBCS for 48 h. The medium was then replaced with F12 supplemented with 0.1% NBCS for 24 h. A cross-shaped wound was generated with a pipette tip in the middle of the well. Cells were washed twice with F12, 0.1% NBCS and then incubated at 37°C in the same medium. The wound area was measured by Image J software (U. S. National Institutes of Health, Bethesda, Maryland, USA), at time 0 (t_0_) and after 24 hours (t_24_). The wound closure was determined by the following equation: [(wound area t_0_ - wound area t_24_)/wound area t_0_]×100. In experiments using conditioned media, 2 mL of media conditioned for 48 h by WT and DS-epi1−/− AoSMCs were added to the culture after the scratch. For migration on pre-deposited matrix, the cells were grown for 48 h in 6-well plates and then removed with 10 mM EDTA in PBS for 10 minutes at 37°C. A small cover glass was placed on the bottom of each well to create the migratory area, and WT and DS-epi1−/− cells from 10% NBCS culture were seeded. The glass was then removed after 6 h leaving a completely cell-free area. At this time, new medium supplemented with 0.1% NBCS was added, and the migration was monitored for 24 h as above.

Cell tracking after wound and measurement of cell speed were performed as follows: AoSMCs were plated into µ-slides 8 wells, ibiTreat (IBIDI, Germany), grown and scratched as above. Time lapse was taken every 10 min using an inverted microscope (Axio Observer.Z1; Zeiss) for 24 hours at 37°C and 5% CO_2_. Individual cells were tracked and analyzed using Volocity 5.0 software (Perkin Elmer) for speed, distance and direction.

### TIRF Microscopy of Adhesion Molecules on the Plasma Membrane

Total internal reflection fluorescence (TIRF) microscopy experiments were performed using an inverted microscope (Axio Observer.Z1; Zeiss) equipped with a Zeiss TIRF module and a 100× 1.46 NA DIC M27 Zeiss TIRF oil-immersion lens and acquisitions were made by using Slidebook 5.5 (3I). Cells were seeded into MatTek dishes (MatTek corporation, MA, USA) and stained overnight with either anti-vinculin (2 µg/ml) or anti-FAK (10 µg/ml). Then the secondary antibody Alexa-546 nm (Invitrogen) was added and finally Phalloidin F-actin staining was performed using 488-phalloidin (A12379; Life Technologies) according to the manufacturer. The extent of co-localization of different markers was analyzed using Image J software and JACoB analysis. Co-localization was measured using the Manders coefficient to evaluate the overlap in fluorescence [21].

### Statistical Analysis

Unpaired Student’s t-tests were used for statistical analyses using GraphPad Prism version 5.01 for Windows and GraphPad Software, San Diego California USA. Statistically significant p values are reported in the figure captions.

## Results

### Reduced IdoA Content Characterizes CS/DS of DS-epi1−/− AoSMCs

**Figure 1:**
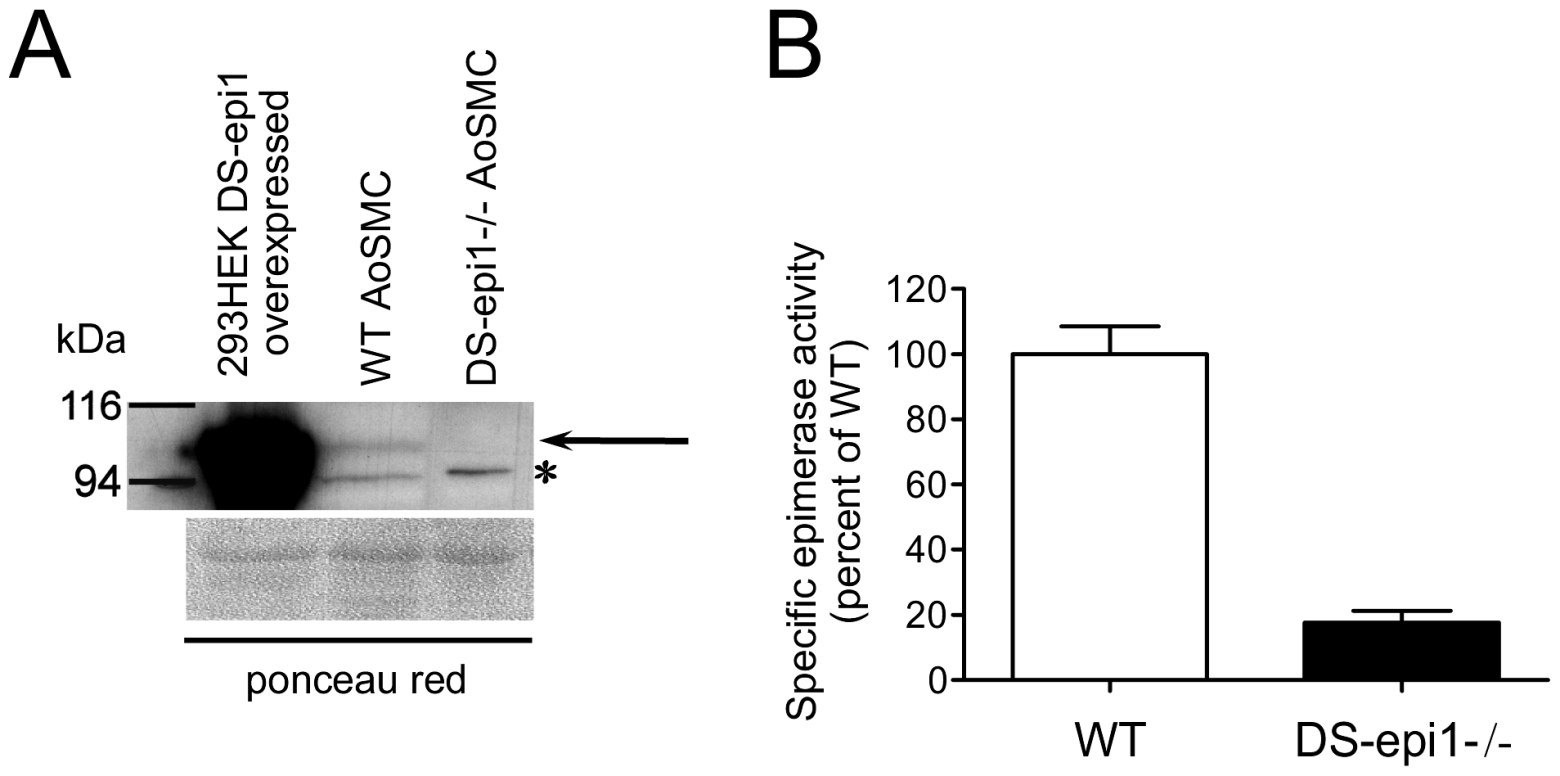
DS-epi1−/− AoSMCs have reduced epimerase activity. A, western blot analysis of 20 µg proteins of total cell lysate. DS-epi1 is visible as a band of around 100 kDa as indicated by the *arrow*. Aspecific band detected both in the WT and in the DS-epi1−/− cell extracts is marked with a *star*. Equal loading is shown by Ponceau Red staining. B, epimerase activity expressed as percentage of WT. Specific epimerase activity was 237 dpm/h/mg for WT AoSMCs. Values are mean ± SEM of triplicates.

**Figure 2:**
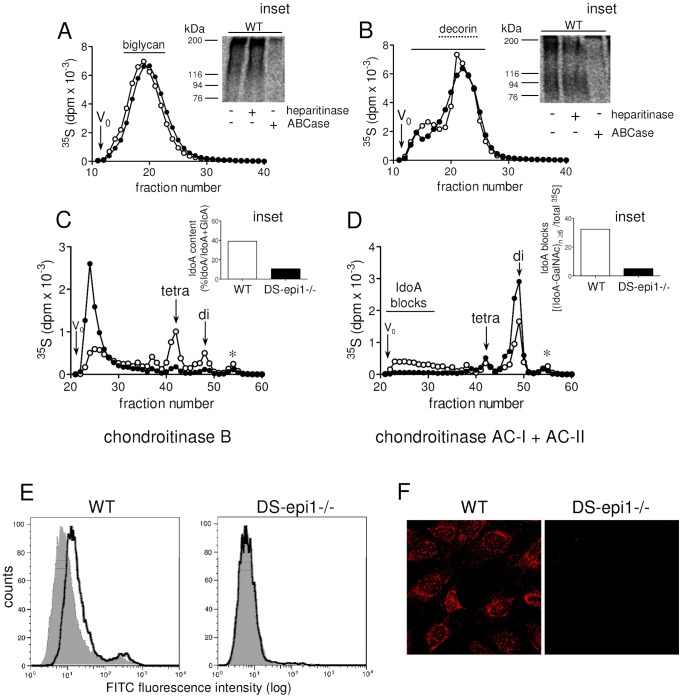
Medium and cell surface DS-epi1−/− CS/DS have reduced IdoA content and lack IdoA-blocks. WT and DS-epi1−/− AoSMCs were labeled with [^35^S] sulfate and PGs released into the medium were isolated on a DE52-column and subsequently analyzed by size exclusion on a Superose 6 column. A-D, WT *open circles*, DS-epi1−/− *filled circles*. Elution profiles of PGs from medium of passage 2 (A) and passage 7 (B) cells are shown. Fractions indicated by a *continuous line* were pooled and analysed on SDS-PAGE after degradation with heparitinase and chondroitinase ABC (Fig. 2A and 2B, insets). Low molecular weight fractions from (B), corresponding to decorin, were pooled as indicated by a *dotted line* and CS/DS was purified. The split products after degradation with chondroitinase B (C) and chondroitinase ACI+ACII (D) were separated on a Superdex Peptide column. Elution positions of di- and tetrasaccharides are indicated by *arrows*; free sulfate peaks, derived from 4-sulfatase contaminating the lyases’ preparations, are indicated by *stars*. Quantification of total IdoA residues from the chromatogram in (C) is shown in the inset. Proportions of IdoA blocks, highlighted in (D) with a *continuous line*, are shown in inset. E, flow cytometric analysis of IdoA-containing epitopes in WT (left panel) and DS-epi1−/− (right panel*)* after cell surface staining of non-permeabilized AoSMCs using the anti-DS antibody GD3A12; negative control (no GD3A12 antibody), *filled profile*. F, confocal immunofluorescence microscopy of IdoA-containing epitopes using the anti-DS antibody GD3A12.

Smooth muscle cells isolated from aortas of WT and DS-epi1−/− mice [18] were used as a cellular model to investigate functions of CS/DS-PGs. WT cells express DS-epi1 as shown by the expected ∼100 kDa band detected in the total cell lysate. The band was absent in the cell lysate of DS-epi1−/− cells (Fig. 1A, arrow). The epimerase activity was measured, and DS-epi1−/− AoSMCs displayed a residual 20% activity (Fig. 1B), which can be ascribed to DS-epi2 [5]. This result is similar to the previously reported residual activity measured in DS-epi1−/− embryonic fibroblasts [22]. To assess the structural variations in the CS/DS chains, WT and DS-epi1−/− cell media were supplemented with ^35^[S]-sulfate and labeled PGs were isolated and analyzed. At identical passages, PGs secreted by WT and DS-epi1−/− cells had similar size. Interestingly, only one PG was predominantly secreted at lower passages (Fig. 2A), whereas at higher passages two peaks were detected (Fig. 2B). SDS-PAGE analysis of native and heparitinase/chondroitinase ABC-treated PGs, pooled as shown in Figures 2A and 2B (black line), revealed that the size of the predominant PGs at different passages was in agreement with biglycan, running between 116 and 200 kDa, and decorin, running between 76 and 116 kDa, (Fig. 2A and B, insets), respectively [23]. At both passages the HS-PGs were a minor component, as seen by the SDS-PAGE after enzymatic treatment. In the following experiments, AoSMCs at higher passages were used. Decorin was isolated and further characterized for CS/DS composition. Purified CS/DS was digested by chondroitinase B, which cleaves GalNAc∼IdoA linkages, or by a mixture of chondroitinase ACI+ACII, which cleave GalNAc∼GlcA linkages. After chondroitinase B digestion, DS-epi1−/− CS/DS chains displayed larger amounts of undigested material than WT chains (Fig. 2C), reflecting reduced IdoA residues. Moreover, the elution profile of WT CS/DS showed large amounts of tetrasaccharides (derived from ∼IdoA-GalNAc-GlcA-GalNAc∼IdoA sequences) and disaccharides (Fig. 2C). The total IdoA content was 39% in WT cells and 11% in DS-epi1−/− CS/DS (Fig. 2C inset). Treatment of WT CS/DS with chondroitinase ACI+ACII resulted in uncleaved material, known as IdoA blocks (Fig. 2D), comprising 32% of total radioactivity in WT chains and 5% in DS-epi1−/− chains (Fig. 2D inset).

The expression of cell surface IdoA was examined by FACS analysis using the GD3A12 phage display antibody recognizing IdoA-blocks containing epitopes [1], [19]. Cell surface IdoA-block structures were undetectable in DS-epi1-deficient cells as shown by the fluorescence profile, which was superimposable on the negative control. On the other hand, the epitopes were present on WT cell surface (Fig. 2E). Additionally, IdoA-blocks containing CS/DS proteoglycans were found on the cell surface of WT (Fig. 2F, left panel) while they were absent in DS-epi1−/− cells (Fig. 2F, right panel), as visualized by confocal microscopy.

### DS-epi1−/− AoSMCs Show a Shifted Composition of Cell Surface HS Epitopes

**Figure 3:**
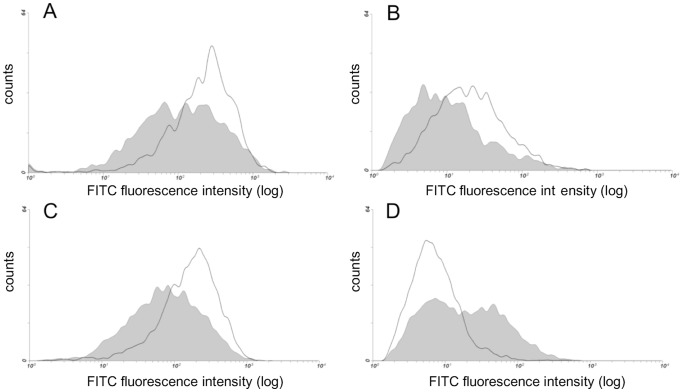
Cell surface HS epitopes change upon functional ablation of DS-epi1. FACS analysis of AoSMCs from WT and DS-epi1−/− mice using a set of anti-HS antibodies. A) mAb-10E4; B) mAb-HepSS-1; C) mAb-NAH46; D) mAb-JM403. The fluorescence distribution profile of WT cells is shown by *empty area*, whereas the one of DS-epi1−/− cells is shown by *shaded area* . The mean value of the fluorescence channel of negative controls (no primary antibody added) was 2. The results shown are from cells at 80–90% confluence. A second experiment carried out on cells at 20–30% confluence gave superimposable results.

To assess whether the aberrant synthesis of DS chains may affect the composition of surface HS chains, we evaluated HS surface epitopes of both WT and DS-epi1−/− AoSMCs. The comparison of HS epitope distribution monitored using a panel of well characterized monoclonal antibodies, revealed differences consistent with changes in HS structure that are secondary to DS-epi1 functional inactivation. In particular, the reactivity to monoclonal antibodies recognizing GlcN-sulfated-containing-epitopes (HepSS-1) [24] (Fig. 3B) or GlcN-acetylated-containing epitopes (NAH46) [25] (Fig. 3C) or both (10E4) [24] (Fig. 3A) was decreased. At the same time the reactivity toward GlcN-unsubstituted-containing epitopes (JM403) [24], [26] (Fig. 3D) was increased. Taken together these results may reveal a shift in HS composition of DS-epi1−/− AoSMCs toward impoverished GlcN-substituted residues and correspondingly enriched GlcN-unsubstituted residues.

### IdoA Influences AoSMC Morphology and Spreading

**Figure 4:**
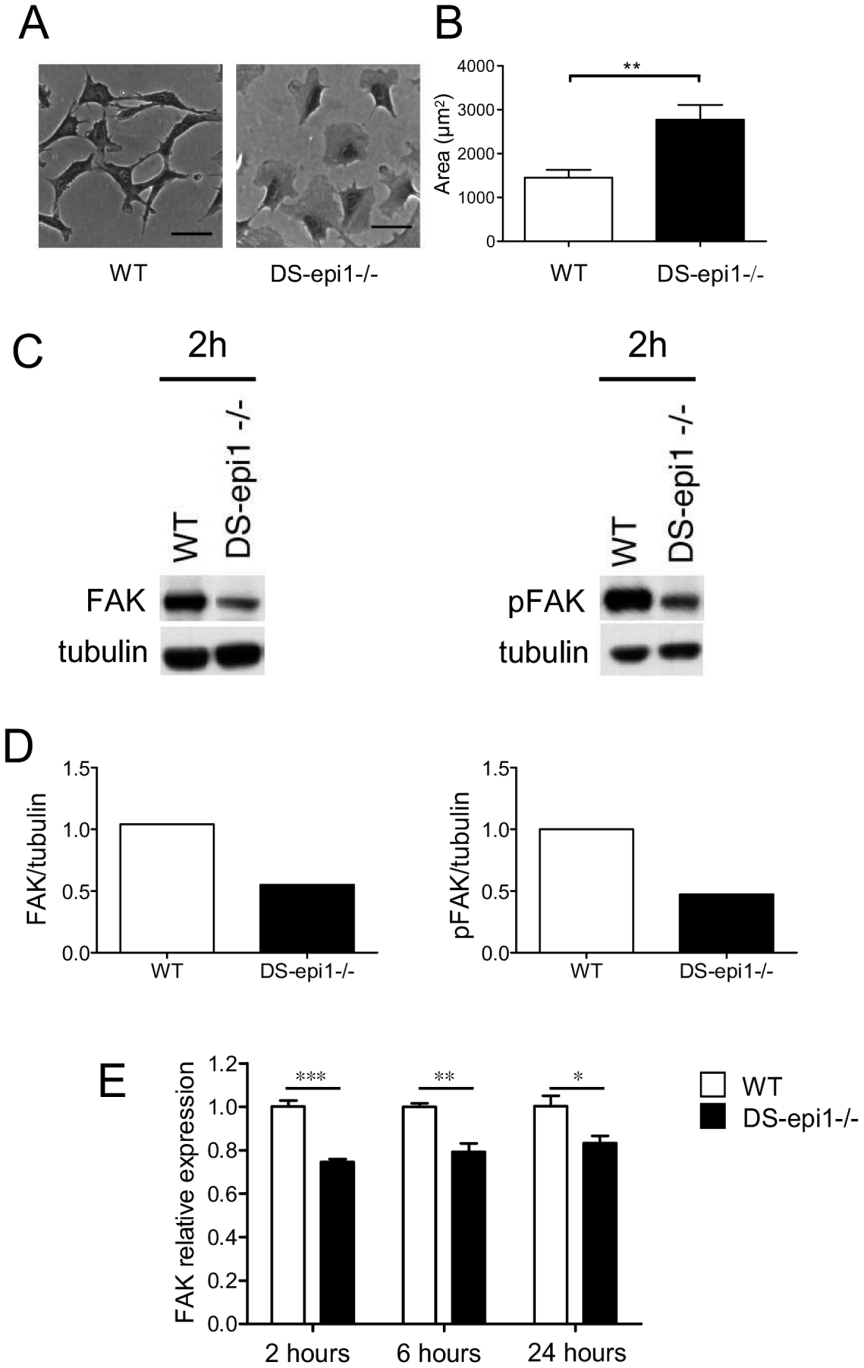
DS-epi1−/− cells are larger and contain less focal adhesion kinases. A, Representative phase contrast image of WT and DS-epi1−/− cells captured 24 h after seeding. B, Calculated cell area from images shown in (A). Data are mean ± SEM (50 total cells of each genotype analysed). **p<0.01. C, Cells were seeded for two hours and harvested, followed by immunoblotting analysis of focal adhesion kinases FAK and pFAK. D, densitometric analyses of immunoblots shown in C. E, FAK mRNA was quantified by qRT-PCR on WT and DS-epi1−/− cells at different times after seeding. The quantification was done in quadriplicates and the experiment was repeated twice with similar results. ***p<0.001, **p<0.05, *p<0.01.

When cultured subconfluently DS-epi1−/− cells had altered morphology, *i.e.* a rounded appearance compared to control cells (Fig. 4A). They were characterized by large flattened protrusions all around the cell body, while WT cells showed an elongated shape with distinct directional protrusions. The surface occupied by the DS-epi1−/− cells was almost twice as large as the WT cells (Fig. 4B). Because focal adhesions are the functional structures responsible for attachment and spreading, some of their components were investigated [27]. DS-epi1-deficient cells had about half as much focal adhesion kinase (FAK) and the activated form, pFAK, as did the WT cells (Fig. 4C and D) 2 hours after seeding. To understand if the effect of decreased IdoA on FAK downregulation was at transcriptional level, qRT-PCR was performed on cells after different times of seeding. FAK mRNA was significantly less in DS-epi1−/− AoSMCs at all time-points examined (Fig. 4E).

### Cell Surface IdoA-containing PGs Affect AoSMC Migration

**Figure 5:**
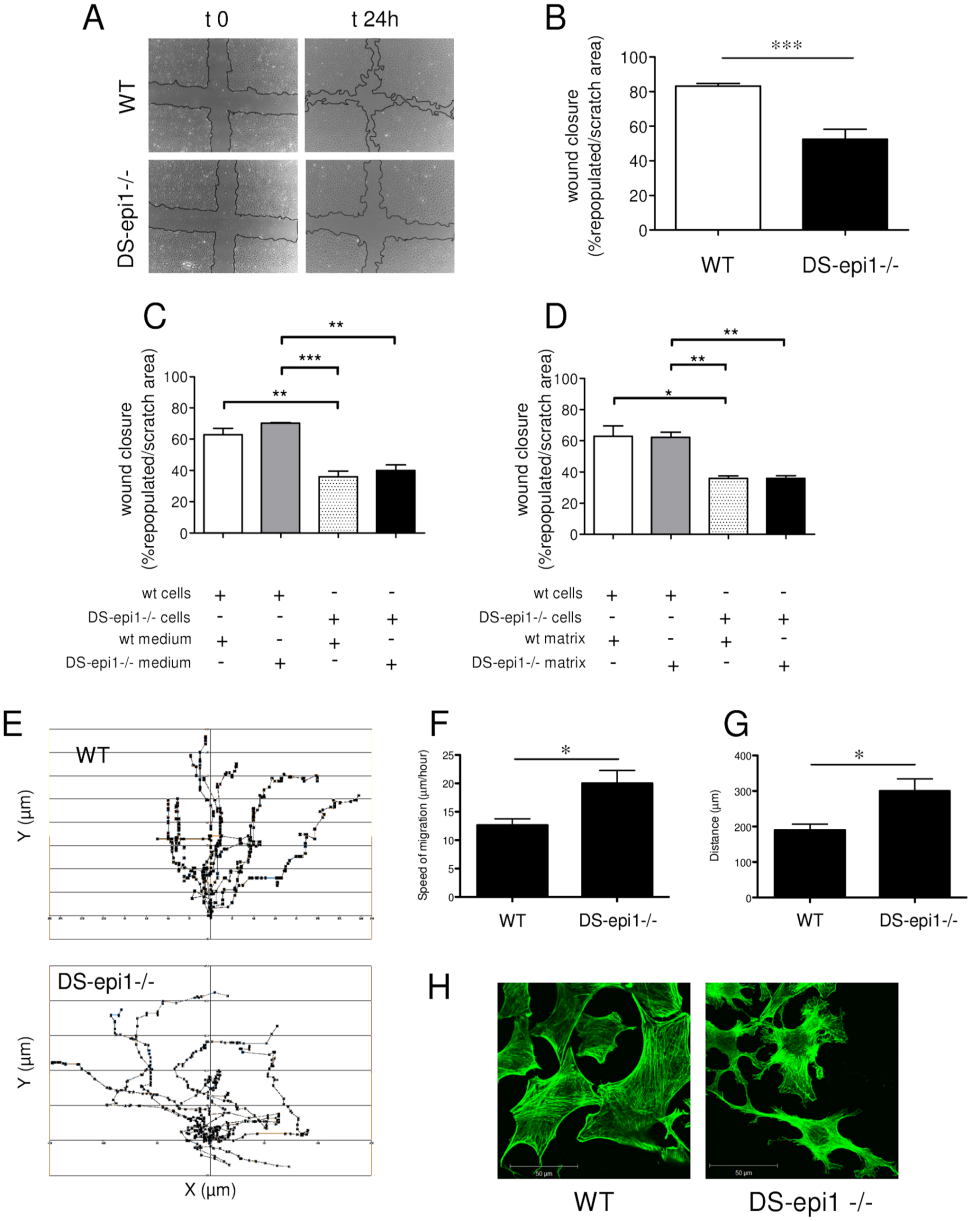
Directional migration is impaired in DS-epi1−/− AoSMCs. Scratch assay was performed (A) and the repopulated area calculated (B). C, migration of WT and DS-epi1−/− cells in the presence of 48 h-conditioned media. D, migration of WT and DS-epi1−/− cells on pre-deposited matrix. C and D: WT cells with WT-conditioned medium/deposited matrix (*white bar*), WT cells with DS-epi1−/− conditioned medium/deposited matrix (*grey bar*), DS-epi1−/− cells with WT-conditioned medium/deposited matrix (*dotted bar*), and DS-epi1−/− cells with DS-epi1−/− conditioned medium/deposited matrix (*black bar*). Data shown are mean ± SEM of triplicates. E, 10 randomly selected cells were individually tracked for 15 hours after scratching and their (F) speed and (G) the distance covered were measured. For each experiment cells with different genotypes were seeded in two chambers and one scratch was made. Three fields/scratch were recorded. Two experiments were made with similar results. H, WT and DS-epi1−/− AoSMCs display an altered cell morphology and cytoskeletal organization during migration. Actin filaments of WT and DS-epi1−/− cells migrating in a scratch assay were stained by phalloidin (green); scale bars 50 µm. *p<0.05 **p<0.01, ***p<0.001).

Since CS/DS-PGs are known to influence cellular properties [17], [20], [28], [29], we investigated proliferation, senescence and migration of our cellular model. DS-epi1−/− cells migrated less in the scratch assay (Fig. 5A). Quantification of repopulated scratched area after 24 hours showed that migration was reduced by 36% in DS-epi1−/− cells compared to WT cells (Fig. 5B). Eventually at longer time-points, and always at 72 hours, the wounded area was completely re-occupied both by WT and DS-epi1−/− AoSMCs.

Cell migration is a complex process partly regulated by the surrounding ECM [30] where IdoA in secreted CS/DS-PGs may play a role. The wound scratch assay was performed in the presence of medium conditioned by WT and DS-epi1−/− cells for 48 h (Fig. 5C). The presence of 10% serum in the conditioned media did not interfere with the assay, since cells did not differentially proliferate (Fig. S1A). Migration of WT cells was unaffected when medium derived from either WT or DS-epi1−/− cells was added. DS-epi1−/− cells displayed equally reduced migration irrespective of the source of the added conditioned medium. To investigate the influence on migration of IdoA in deposited ECM, 48 h cultured cells were removed with EDTA. WT and DS-epi1−/− cells were then seeded on WT or DS-epi1−/− matrices and migration assays were carried out. Migration of DS-epi1−/− cells was impaired independently of the ECM type (Fig. 5D). In summary, these results demonstrated that migration is not affected by a decrease of IdoA in soluble or in ECM-deposited CS/DS-PGs. Consequently, the reduced migration of DS-epi1−/− cells is most likely due to IdoA reduction in CS/DS-PGs expressed on the cell surface. No differences in proliferation and senescence were observed comparing the two genotypes (Fig. S1A and B).

### DS-epi1−/− AoSMCs have Reduced Directional Migration, an Altered Morphology and Cytoskeletal Organization

Trying to elucidate the primary defect in migration, the same wounded fields were recorded every ten minutes for 24 hours. Again, the delayed capacity of DS-epi1−/− AoSMCs in re-populating the empty space was visually confirmed. Individual cells were tracked for 15 hours and it was apparent that the WT cells moved mainly perpendicular to the scratch towards the other side, while the DS-epi1−/− cells moved mainly parallel to the scratch or along small or big circles (Fig. 5E and Movies S1 and S2, showing movies after scratch). DS-epi1−/− AoSMCs were actually faster (Fig. 5F) and therefore moved a longer distance (Fig. 5G) than control cells. In summary, DS-epi1−/− cells lost the directionality of migration. Phalloidin staining of actin stress fibers of migrating cells was performed 24 hours after the scratch. WT AoSMCs displayed a polygonal shape with stress fibers spanning the whole cell (Fig. 5H). In contrast, DS-epi1−/− cells displayed a different cellular morphology with branched protrusions and actin fibers radially disposed around the nucleus and towards the protrusions.

### In DS-epi1−/− AoSMCs Stress Fibers are Increased in the Proximity of the Plasma Membrane Adhering to Substratum and Focal Adhesion Sites are Decreased

**Figure 6:**
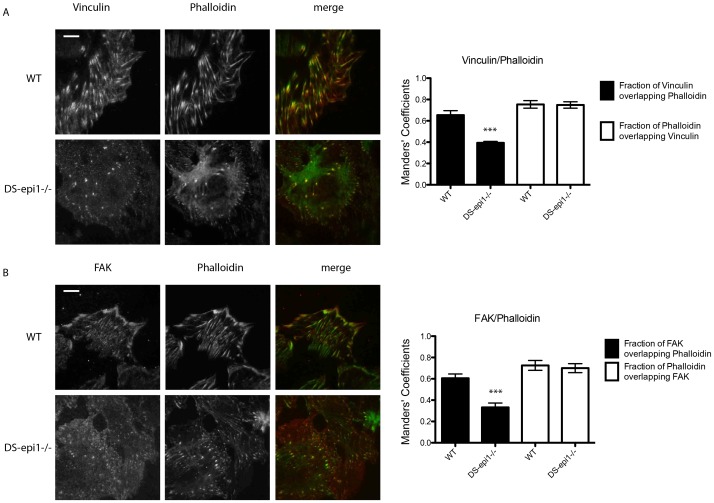
Focal adhesion sites are decreased in the DS-epi1−/− cell membrane attached to substratum. Total internal reflection fluorescence (TIRF) images were taken after (A, left panel) vinculin/phalloidin or (B, left panel) FAK/phalloidin double staining. Scale bars = 10 µm. Overlapping sites were measured with Manders’ coefficients in randomly chosen 10 cells (A and B, right panels). The staining experiments were repeated three times with similar results. ***p<0.001.

Total internal reflection fluorescence (TIRF) microscopy allows visualization of approx. 100 nm thick section of the cell adjacent to the substratum, which mainly includes cell membrane. TIRF images showed increased phalloidin staining in DS-epi1−/− cells (Fig. 6A, left panel). Focal adhesion sites, indicated as the sites of co-localization of either FAK or vinculin with phalloidin staining (yellow signal in the figures), are decreased in mutated cells compared to control, as measured with the Manders’ coefficients (Fig. 6A and B, right panels). As control that the degree of co-localization is not dictated by the increased stress fibers observed in mutated cell, the fraction of phalloidin signal overlapping FAK or vinculin signals were unchanged in control and mutated cells.

## Discussion

In this report we show that, following DS-epi1 ablation, an IdoA decrease in CS/DS on the cell surface results in changed spreading and diminished directional migration of AoSMCs. DS-epi1−/− AoSMCs did not lose the capability to migrate, and indeed migrated faster, but had impaired movement perpendicular to the wounded area in the direction of the source of chemotactic factors.

In a previous paper we reported that a DS-epi1-downregulated cancer cell line had reduced migratory ability and aberrant adhesion and spreading [1]. As cancer cells are *per se* altered, it was important to analyze migration using non tumorigenic cells such as AoSMCs obtained from WT and DS-epi1−/− mice [18], a model that can better exemplify cellular mechanisms occurring *in vivo*. It was described that fibroblasts lacking the HS-epimerase, and therefore lacking IdoA in heparan sulfate, migrate less in response to FGF-2 [31]. Similarly, we found that long stretches of IdoA (IdoA blocks) in CS/DS regulate migration. This effect was studied in the presence of 10 or 0.1% serum. We cannot exclude that in a context of stimulation by a specific growth factor, decreased IdoA residues may result in a more pronounced migration defect, as seen in cancer cells with HGF [1]. PDGF-BB and activated EGFR are important growth and chemotactic signals for smooth muscle cells [32]. CS/DS binds to PDGF-BB [33], [34] and to the EGFR ligand HB-EGF [35]. In addition, cell surface CS/DS-PGs bind the EGF-like domain in ERM receptor which is an EGF receptor restricted to leukocytes and smooth muscle cells [36]. PDGF-BB induces in vascular smooth muscle cells directional migration [37] and the asymmetry of phospho-EGFR signaling is essential for proper migration and directional persistence of myofibroblasts [38]. It would be important to identify in future studies the chemotactic factor(s) whose activity is impaired due to decreased iduronic acid in CS/DS chains.

The strength of this present study is that the influence of IdoA in CS/DS is studied under native conditions. In most of the reports CS/DS-PGs and CS/DS free chains were exogenously added [39], [40] or removed by enzymatic digestion [20], showing contradictory results. For instance, decorin was shown to slow the migration rate of osteosarcoma cells by a mechanism depending on its CS/DS chain [28], while CS/DS-PGs biglycan and decorin, independently of their CS/DS, increased mobility of lung fibroblasts [39]. Moreover, addition of low molecular weight CS/DS stimulated migration of endothelial cells [40]. We could establish that CS/DS-PGs, either supplied in conditioned media or in pre-deposited ECM, failed to alter the motility of WT and DS-epi1−/− cells, which indicates that the mechanism behind the change of migration and adhesion is related to cell surface CS/DS-PGs. Thus, IdoA is an essential component of cell membrane structures needed for the correct cell organization during migration.

The observed fine structural changes in HS are interesting, even if no quantitative conclusions can be drawn by the immunological epitope detection approach. The data raise the possibility that a cross-talk exists between the DS and HS metabolic pathways. On the opposite, lack of HS epimerase did not change the amount and structure of CS/DS released into the medium by mouse embryonic fibroblasts [31].

DS-epi1−/− AoSMCs showed an aberrant stress fibers organization both in the whole cell (Fig. 5H) and in the cell membrane attached to the substratum (Fig. 6A and B). In addition, total cellular protein and mRNA amount of FAK and protein pFAK were lower than in WT cells. FAK plays a central role both in regulating adhesion and migration. FAK and pFAK regulate the recruitment and subsequent activation of a number of intracellular signals important for cytoskeleton rearrangement [27], [41], and it is not surprising that a perturbation of such a system influences cell shape and motility. Cell membrane PGs are required for correct cytoskeletal organization as recently described in syndecan-4 KO fibroblasts [42]. Cell-bound IdoA-containing CS/DS-PGs might be involved in stabilization of membrane micro domains which in turn transfer signals to the inner compartment. NG2, syndecan-1,-3 and -4, betaglycan, neuropilin-1, CD44 and integrin α_5_β_1_ are all CS/DS-PGs present at the cell surface, but only syndecan-1 and CD44 unequivocally contain IdoA residues [2], [11], [14]. Further investigation regarding IdoA organization in all these PGs is therefore required. Integrin α_5_β_1_ has been described as a part-time PG, with no specific characterization of its CS/DS chain [13]; it is involved in functional adhesion to the substrate, modulation of PG effect on intracellular signaling [30] and FAK phosphorylation [43]. NG2 is expressed by mouse AoSMCs, and its role in migration has been explored [44], although no specific attention has been pointed to its CS/DS chain [12]. CD44 IdoA structures have been claimed to be essential for fibroblast invasion into fibronectin/fibrin gels [11]. Moreover, reports have shown that CD44 is localized in the focal adhesions of invadopodia [45], anchoring the cytoskeleton elements [46], and concentrating metalloproteases [47], making its CS/DS chain a candidate for regulation of both focal adhesion complexes and organization of actin stress fibers.

In conclusion, our data show that IdoA reduction alters the directional persistence of migrating AoSMCs. In molecular terms, FAK levels were decreased and stress fibers were increased at the attachment sites, accompanied by dysregulation of focal adhesion sites which were abnormally distributed. Smooth muscle cell migration is of critical importance in both normal and pathological conditions such as atherosclerosis [48]. Modulation of IdoA expression could potentially be a new therapeutic avenue for the treatment of such disease, reducing vessel thickening, and thus delaying the onset and development of the atherosclerotic plaque.

## Supporting Information

Figure S1 **Proliferation and senescence of AoSMCs are not affected by DS-epi1 deficiency.** A, proliferation assay. Cells were stained with crystal violet and the optical density was determined at 595 nm after different days in culture. B, senescence staining, i.e. β–galactosidase staining. No specific blue staining is seen in the cytoplasm of either WT or DS-epi1−/− AoSMCs.
(TIF)

Methods S1 **Proliferation and senescence assay.** WT and DS-epi1−/− AoSMC were seeded in 96-well plates at a concentration of 1000 cells/cm^2^. Cells were starved in 0.2% NCBS supplemented medium for 24 h, followed by addition of complete medium (10% NCBS) and incubated for 24 h, 48 h or 72 h. Cells were stained with crystal violet as reported in [20]. The senescence assay (CS0030; Sigma) was performed according to the manufacturer.
(DOC)

Movie S1 **WT cells display directional migration. Movement of WT cells was recorded for 15 hour after the scratch was made.** Cells moved preferentially toward one direction, i.e. the bottom of the panel, where the scratch was made.
(AVI)

Movie S2 **DS-epi1−/− cells display altered directional migration. Movement of DS-epi1−/− cells was recorded for 15 hour after the scratch was made.** Cells moved mainly parallel to the scratch or along small or big circles.
(AVI)
