# Supplementary figures and images for: Iduronic Acid in Chondroitin/Dermatan Sulfate Affects Directional Migration of Aortic Smooth Muscle Cells
Source: PLoS One. 2013 Jul 2;8(7):e66704. doi: 10.1371/journal.pone.0066704 (PMC3699603; doi:10.1371/journal.pone.0066704)

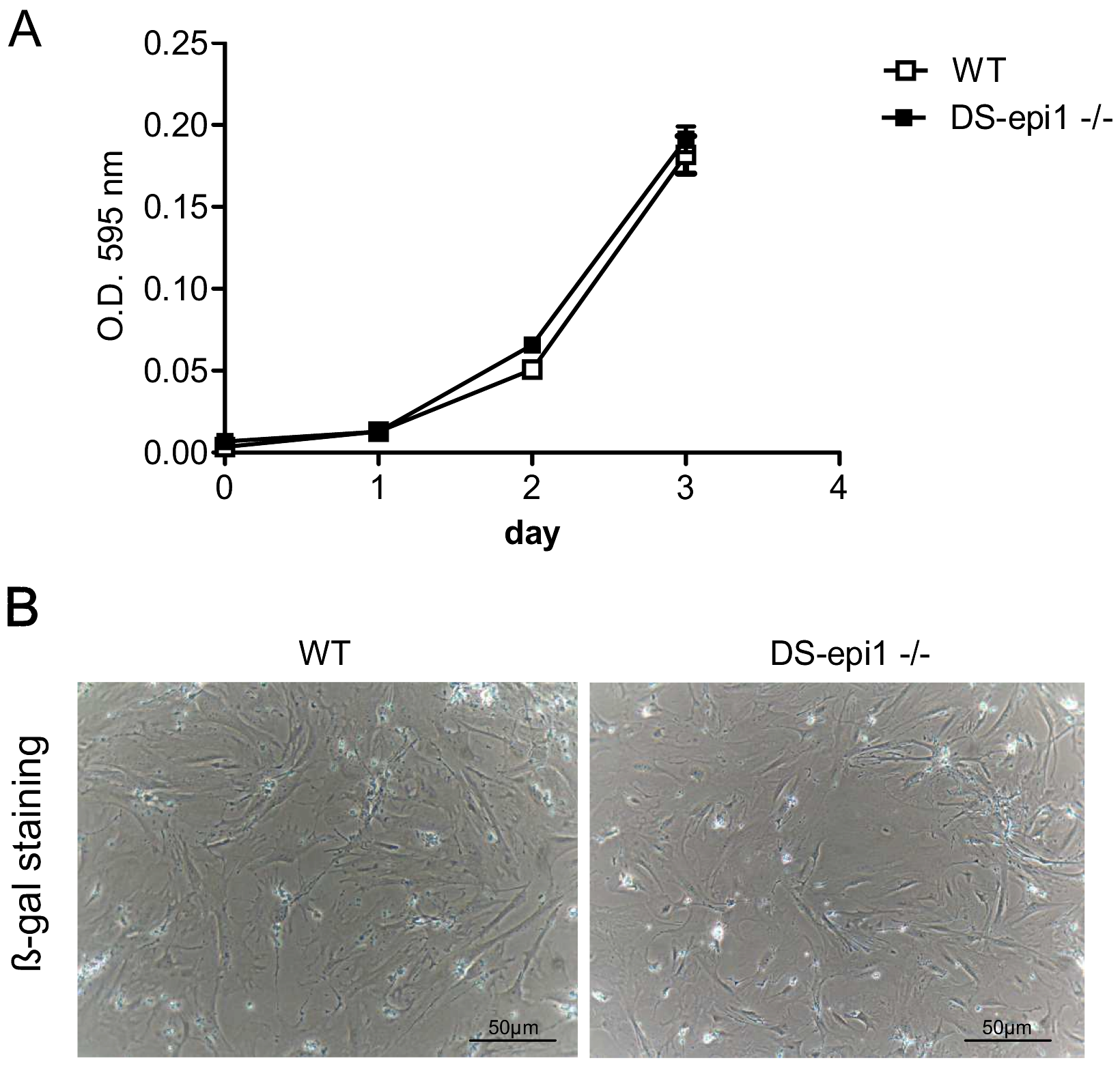

Supplement: Figure S1 — Proliferation and senescence of AoSMCs are not affected by DS-epi1 deficiency. A, proliferation assay. Cells were stained with crystal violet and the optical density was determined at 595 nm after different days in culture. B, senescence staining, i.e. β–galactosidase staining. No specific blue staining is seen in the cytoplasm of either WT or DS-epi1−/− AoSMCs. (TIF) [file pone.0066704.s001.tif]
