# Supplementary material for: Iduronic Acid in Chondroitin/Dermatan Sulfate Affects Directional Migration of Aortic Smooth Muscle Cells
Source: PLoS One. 2013 Jul 2;8(7):e66704. doi: 10.1371/journal.pone.0066704 (PMC3699603; doi:10.1371/journal.pone.0066704)
Supplement: Methods S1 — Proliferation and senescence assay. WT and DS-epi1−/− AoSMC were seeded in 96-well plates at a concentration of 1000 cells/cm2. Cells were starved in 0.2% NCBS supplemented medium for 24 h, followed by addition of complete medium (10% NCBS) and incubated for 24 h, 48 h or 72 h. Cells were stained with crystal violet as reported in [20]. The senescence assay (CS0030; Sigma) was performed according to the manufacturer. (DOC) [file pone.0066704.s002.doc]

**Supporting information**

**Methods**

**Proliferation and senescence assay**

WT and DS-epi1-/- AoSMC were seeded in 96-well plates at a concentration of 1000 cells/cm^2^. Cells were starved in 0.2% NCBS supplemented medium for 24 h, followed by addition of complete medium (10% NCBS) and incubated for 24 h, 48 h or 72 h. Cells were stained with crystal violet as reported in [20]. The senescence assay (CS0030; Sigma) was performed according to the manufacturer.
